# Decellularized Human Dermal Matrix as a Biological Scaffold for Cardiac Repair and Regeneration

**DOI:** 10.3389/fbioe.2020.00229

**Published:** 2020-03-20

**Authors:** Immacolata Belviso, Veronica Romano, Anna Maria Sacco, Giulia Ricci, Diana Massai, Marcella Cammarota, Angiolina Catizone, Chiara Schiraldi, Daria Nurzynska, Mara Terzini, Alessandra Aldieri, Gianpaolo Serino, Fabrizio Schonauer, Felice Sirico, Francesco D’Andrea, Stefania Montagnani, Franca Di Meglio, Clotilde Castaldo

**Affiliations:** ^1^Department of Public Health, University of Naples Federico II, Naples, Italy; ^2^Department of Experimental Medicine, Università degli Studi della Campania Luigi Vanvitelli, Naples, Italy; ^3^Department of Mechanical and Aerospace Engineering, Politecnico di Torino, Turin, Italy; ^4^Department of Anatomy, Histology, Forensic-Medicine and Orthopedics, Sapienza University of Rome, Rome, Italy

**Keywords:** decellularized extracellular matrix, human dermal matrix, cardiac tissue engineering/regenerative medicine, human cardiac progenitor cells, biological scaffolds

## Abstract

The complex and highly organized environment in which cells reside consists primarily of the extracellular matrix (ECM) that delivers biological signals and physical stimuli to resident cells. In the native myocardium, the ECM contributes to both heart compliance and cardiomyocyte maturation and function. Thus, myocardium regeneration cannot be accomplished if cardiac ECM is not restored. We hypothesize that decellularized human skin might make an easily accessible and viable alternate biological scaffold for cardiac tissue engineering (CTE). To test our hypothesis, we decellularized specimens of both human skin and human myocardium and analyzed and compared their composition by histological methods and quantitative assays. Decellularized dermal matrix was then cut into 600-μm-thick sections and either tested by uniaxial tensile stretching to characterize its mechanical behavior or used as three-dimensional scaffold to assess its capability to support regeneration by resident cardiac progenitor cells (hCPCs) *in vitro*. Histological and quantitative analyses of the dermal matrix provided evidence of both effective decellularization with preserved tissue architecture and retention of ECM proteins and growth factors typical of cardiac matrix. Further, the elastic modulus of the dermal matrix resulted comparable with that reported in literature for the human myocardium and, when tested *in vitro*, dermal matrix resulted a comfortable and protective substrate promoting and supporting hCPC engraftment, survival and cardiomyogenic potential. Our study provides compelling evidence that dermal matrix holds promise as a fully autologous and cost-effective biological scaffold for CTE.

## Introduction

Accounting for more than 13% of all deaths, ischemic heart disease (IHD) is the single most common cause of death globally (GBD 2016 Causes of Death Collaborators, 2017). Although IHD is preventable by addressing behavioral risk factors such as tobacco use and harmful use of alcohol, unhealthy diet, physical inactivity, and obesity, it has remained the leading cause of death for the last 16 years worldwide (GBD 2016 Risk Factors Collaborators, 2017). IHD is most commonly caused by the formation of a thrombus on a ruptured atherosclerotic plaque in the wall of a coronary artery. Blocking the blood flow, the clot prevents the supply of oxygen and nutrients to the myocardium fed by the involved vessel and leads to the ischemic death of cardiac myocytes. Since the heart has very limited regenerative ability, the reparative process that takes place after myocardial damage is characterized by the replacement of dead myocytes and the microenvironment they reside with non-compliant scar tissue. The complex and highly organized environment in which cells reside *in vivo* consists mostly of the extracellular matrix (ECM) that results from a tissue-specific combination of structural and functional proteins, polysaccharides and soluble factors (Brown and Badylak, 2014). The composition of the ECM and the exact spatial orientation of its components is responsible for both the biological signaling delivered to resident cells and the generation and transfer of mechanical forces to which cells are exposed (Frantz et al., 2010; Wade and Burdick, 2012). Therefore, the profound changes in the ventricular wall structure and mechanics caused by the ischemic damage impact upon cardiac function. Currently, the only effective therapeutic option capable of restoring the physiological heart function is heart transplant. However, due to organ donor shortage and to the high incidence of IHD, alternative strategies are urgently needed. Cardiac tissue engineering (CTE) aiming at developing three-dimensional myocardium-like scaffolds for therapeutic use by combining cells, synthetic or natural biomaterials, and biomimetic signals is rapidly emerging as the most promising alternative to heart transplant (Vunjak-Novakovic et al., 2010; Massai et al., 2013; Feric and Radisic, 2016; Stoppel et al., 2016; Fujita and Zimmermann, 2017; Weinberger et al., 2017; Madonna et al., 2019). Among biomaterials, decellularized tissues are emerging as the most promising scaffolds for regenerative medicine, due to their potential to provide natural biological cues (Yaling et al., 2016). However, the regenerative capability of the substrate depends not only on its chemical properties, but also on its ability to recapitulate the mechanical behavior of the native tissue, providing physical signals essential for mechanotransduction pathways (Huang et al., 2004; O’Brien, 2011). Specifically, in native myocardium, heart compliance and cardiomyocyte alignment and differentiation are strictly dependent on the elastic properties of the tissue. In particular, the content of elastin, the dominant mammalian elastic protein and the main component of elastic fibers (Kielty et al., 2002; Li et al., 2014), has been related to cardiac myocyte differentiation and maturation as it progressively increases in the developing mammalian heart, but then decreases soon after birth (Hanson et al., 2013; Thimm et al., 2015). Interestingly, elastin-based biomaterials have been developed and used to enhance tissue biocompatibility and promote stem cell differentiation (Celebi et al., 2012; Ozsvar et al., 2015). In this perspective, due to its remarkable intrinsic elasticity and to the anisotropic mechanical behavior (Terzini et al., 2016), the skin ECM might recapitulate, at least partially, the mechanical properties of the myocardium (Gilbert et al., 2007). Furthermore, while in the native myocardium elasticity is essentially conferred by the cardiomyocytes themselves (Jacot et al., 2010) and, thus, the decellularization process leads to scaffolds with poor mechanical properties, skin elasticity is provided by the ECM rather than by the resident cells and, then, the decellularization treatment is unlikely to cause loss of elasticity (Hoganson et al., 2010; Krieg and Aumailley, 2011). On this basis, we hypothesize that dermal decellularized ECM (d-ECM) may represent an innovative and viable alternate biological scaffold to restore myocardial microenvironment for future *in vitro* cardiac tissue production. To test our hypothesis, we collected specimens of adult human skin from patients undergoing abdominoplasty and prepared scaffolds of decellularized human skin (d-HuSk). The composition of d-HuSk and its potential to deliver biological signals comparable to that of the cardiac native matrix were evaluated by histochemistry and immunohistochemistry analyses and by specific quantitative assays. Additionally, uniaxial tensile tests were performed to mechanically characterize d-HuSk. Finally, 600-μm-thick sections of d-HuSk were seeded with resident human cardiac progenitor cells (hCPCs) to test their ability to ensure hCPC engraftment and survival and to support hCPC retention of the expression of markers specific for cardiac myocytes.

## Materials and Methods

### Tissue Specimens

Skin specimens were obtained from patients undergoing abdominoplasty (*n* = 8, mean age 42.25 ± 7.94). Upon receipt, subcutaneous tissue was removed, and multiple samples were cut marking Langer’s line orientation (Langer, 1978). Resulting specimens were then promptly processed for histological analysis or decellularization. Further, triplets of paired specimens weighting 20 mg each were collected for each patient for a comparative analysis between native and decellularized skin in terms of elastin and glycosaminoglycan (GAG) content, and the samples were snap frozen until use. Cardiac specimens were harvested from macroscopically uninjured areas of the left ventricle of explanted hearts of patients undergoing heart transplant (*n* = 10, mean age 49.5 ± 4.7) and snap-frozen or enzymatically digested to isolate hCPCs. Patients provided written informed consent and specimens were collected without patient identifiers, following protocols approved by the Hospital Ethical Committee and in conformity with the principles outlined in the Declaration of Helsinki.

### Decellularization

Following the simple and effective protocol we recently described for the decellularization of human myocardial sections (Di Meglio et al., 2017), skin specimens and 600-μm-thick sections of human myocardium were incubated in decellularizing solution for 24 h. Decellularization of skin specimens was obtained by placing them in a beaker containing the decellularizing solution under constant stirring, while sections of human myocardium were decellularized (d-HuM) under constant agitation on an orbital shaker. The solution was replaced every 8 h. During the decellularization of skin specimens, and more often after 16 h, the epidermis detached from the dermis and was removed. d-HuSk and d-HuM samples were then rinsed for 24 h in antibiotic solution and then for an additional 30 min in sterile bidistilled water. After decellularization, samples of d-HuSk were either fixed in formalin for paraffin embedding and histological analysis or stored at −80°C until use for molecular analysis and cryosectioning. Sections of d-HuM were either stored at 4°C in Ham’s F12 medium (Sigma-Aldrich) supplemented with 10% FBS (Sigma-Aldrich), 5% horse serum (Sigma-Aldrich), 10 ng/ml basic fibroblast growth factor (bFGF) (Peprotech, Rocky Hill, NJ, United States), and 50 IU/ml penicillin G-streptomycin (Sigma-Aldrich) (F12K medium), or stored in dry conditions at −80°C until use.

### Cryosectioning of Decellularized Dermal Matrix

As previously described for human myocardium (Di Meglio et al., 2017), frozen specimens of d-HuSk were mounted on a cryostat chuck using Tissue Freezing Medium (Leica Microsystems, Wetzlar, Germany) and sliced into 600-μm-thick sections by a Leica CM1950 cryostat (Leica Microsystems). Cryosections were collected in sterile cell culture dishes and stored at 4°C in the same medium described for d-HuM sections until use.

### Quantitative Measurement of DNA Content

Genomic DNA (gDNA) was extracted from specimens of frozen native skin (*n* = 4) and d-HuSk (*n* = 6), using the AllPrep DNA/RNA Mini Kit (Qiagen, Hilden, Germany), according to the manufacturer’s instructions. DNA concentration was determined by measuring the absorbance at 260 nm using a Nanodrop1000 (Thermo Scientific, Waltham, MA, United States) and the gDNA band was visualized with agarose electrophoresis. Data were averaged and expressed as the mean ng of DNA ± SEM per mg of dry tissue.

### Histochemistry and Immunohistochemistry

Specimens of native skin (*n* = 8) and d-HuSk (*n* = 8) were fixed in 10% neutral-buffered formalin, dehydrated in a graded series of alcohols, embedded in paraffin, then sliced into serial 5-μm-thick sections. Following standard protocols, after rehydration sections were stained with Hematoxylin and Eosin to evaluate the effectiveness of decellularization, and with Paraldehyde fuchsin Gomori or Alcian blue to detect elastic fibers or GAGs, respectively, by specific staining kits (both from Bio-Optica, Milan, Italy). For the immunodetection of collagen, fibronectin, tenascin, and laminin, sections of d-HuSk were deparaffinized, rehydrated, and immunostained using indirect immunoperoxidase technique. The presence and localization of antigen ⋅ antibody complexes were revealed by the UltraVision LP Detection System HRP Polymer & DAB Plus Chromogen (Thermo Scientific, Waltham, MA, United States), as previously described (Di Meglio et al., 2017). Stained sections were evaluated and documented by at least three independent observers using a light microscope DM2000 Led (Leica Microsystems) equipped with an ICC50HD camera (Leica Microsystems).

### Quantitative Measurement of Elastin

Elastin was extracted from paired specimens of native skin (*n* = 5) and d-HuSk (*n* = 5) and from frozen specimens of d-HuM (*n* = 5), weighting 20 mg each before decellularization. Following manufacturer’s directions, elastin was extracted heating specimens at 100°C for three 1-h periods in 0.25 M oxalic acid. Tissue extracts were then assayed in the Fastin Elastin Assay quantitative dye-binding method (Biocolor, Ltd., Carrickfergus, United Kingdom), in accordance with manual provided by manufacturer. Briefly, samples were incubated with Elastin Precipitating Reagent to obtain the complete precipitation of α-elastin, to which Dye Reagent was then added. Elastin-Dye complexes that formed were treated with Dye Dissociation Reagent before proceeding with absorbance reading. All samples were tested in triplicate and the absorbance was read at 490 nm using the ELx800 Absorbance Microplate Reader (BioTek Instruments, Winooski, VT, United States), and analyzed with Microsoft Excel (Microsoft Corp., Redmond, WA, United States) to generate the calibration curve and calculate the final concentration of elastin for each absorbance reading. Data were averaged and expressed as the mean value ± SEM of μg of elastin per mg of dry tissue.

### Quantitative Measurement of Sulfated GAGs (sGAGs)

As described for the Fastin Elastin Assay, frozen samples of 20 mg each of native skin (*n* = 5) and frozen samples of d-HuSk (*n* = 5) and d-HuM (*n* = 5) weighting 20 mg each before decellularization were assayed for the quantitative analysis of sGAG content in Blyscan Assay (Biocolor, Ltd.). All samples were tested in triplicate. Assay was performed according to previously published protocol (Di Meglio et al., 2017). Absorbance was read at 600 nm using the BioPhotometer (Eppendorf) and analyzed with Microsoft Excel (Microsoft Corp.) to generate the calibration curve and calculate the final concentration of sGAGs for each absorbance reading. Data were averaged and expressed as the mean value ± SEM of μg of sGAGs per mg of dry tissue.

### Growth Factor Array

Frozen d-HuSk (*n* = 4) and d-HuM (*n* = 4) specimens were lysed as previously described (Di Meglio et al., 2017). Protein concentration was determined by a Bradford assay, and then 100 μg of each tissue lysate was assayed in the Human Growth Factor Array C1 (Raybiotech, Norcross, GA, United States). The assay and following analysis were performed as previously described (Di Meglio et al., 2017). Data were expressed as the mean ± SEM.

### Mechanical Characterization

The mechanical behavior of d-HuSk was characterized by uniaxial tensile stretching. For three donors, 3 to 8 cryosections were cut along and across the Langer’s lines with a custom-made die cutter, and stored in 50 ml sterile plastic tubes in saline solution at 4°C. The specimens were measured by photogrammetry, using a digital camera (EOS 5D Mark II, Canon Inc., Tokyo, Japan), with an autofocus lens for macro photography (EF 100 mm f/2.8 Macro USM, Canon Inc.), mounted on a stand. The measuring process was performed by ImageJ software^1^. The average width of the specimens resulted in 4.80 ± 0.84 mm, while thickness, set during the cryosectioning, was assumed to be equal to 600 μm. To characterize the specimens in room-temperature and “wet-like” conditions, the plastic tubes were taken out of the fridge 1 h before the test, and each specimen was collected just before testing. Uniaxial tensile tests were carried out with a universal testing machine (QTest/10, MTS Systems Corporation, Eden Prairie, Minnesota, United States), and specimens were clamped in titanium grips with knurled-flat faces, specifically developed for preventing specimen slipping (TA Instruments, Inc., New Castle, DE, United States). The grips were placed at an initial distance of 5 mm, and the strain rate was set equal to 3.2% s^–1^ (Terzini et al., 2016). The results of uniaxial tensile tests were reported in terms of engineering stress and strain. The engineering stress σ was calculated as:

$$\sigma =\frac{F}{A_{0}}$$

where *F* is the recorded applied force, and *A*_0_ is the original cross-sectional area of the specimen. The engineering strain ε, referred to the increasing distance between the grips, was expressed as:

$$\varepsilon =\frac{\Delta \,L}{L_{0}}$$

where Δ*L* is the change in length, and *L*_0_ is the original length of the specimen. From the engineering stress-strain curves, the stiffness of the specimens was measured as the elastic modulus at increasing levels of strain. In particular, considering the human left ventricular longitudinal strain range (Kuznetsova et al., 2008), the elastic moduli at 10% (*E*_10__%_) and 20% (*E*_20__%_) of strain were computed as the stress-strain curve slope at 10 and 20% of strain, respectively. The average values of the measured elastic moduli were then compared with literature data on human myocardium (Jawad et al., 2007; Chen et al., 2008; Bouten et al., 2011; Sommer et al., 2015; Terzini et al., 2016). In addition, to assess the tissue rupture behavior, the ultimate tensile strength (UTS, i.e., the maximum stress achieved by the stress-strain curve) and the ultimate strain (ε_*UTS*_, i.e., the strain at which the UTS occurs) were investigated. The method applied to extract UTS and ε_*UTS*_ is described in detail in Section “Supplementary Material” and Supplementary Table S1.

### Cell Culture

To isolate hCPCs, myocardial specimens were dissected, minced, and enzymatically disaggregated as previously described (Nurzynska et al., 2013). Briefly, samples were digested with 0.25% trypsin and 0.1% collagenase (both from Sigma-Aldrich) and the obtained cell population was depleted of fibroblasts by incubation with anti-fibroblast MicroBeads (Miltenyi Biotec, Bergisch Gladbach, Germany), to magnetically label fibroblasts, and then loading cells onto a MACS column (Miltenyi Biotec) placed in the magnetic field of a MACS separator (Miltenyi Biotec) to retain labeled fibroblasts within the column and allow unlabeled cells to run through. Hence, hCPCs were purified from the negative cell fraction by positive selection with anti-human-CD117 MicroBeads. The so obtained hCPCs were then collected and used for repopulating d-HuSk and d-HuM to evaluate d-HuSk cytocompatibility and ability to support hCPC engraftment and differentiation potential.

### *In vitro* Assay of Decellularized Dermal Matrix Biocompatibility

Six hundred-micrometer-thick cryosections of d-Husk (*n* = 3) were cut and placed on sterile 96-well culture plates, sterilized by exposure to ultraviolet radiation for 40 min, then rehydrated for 7 days with F12K medium in an incubator at 37°C with 5% CO_2_. Successively, 5 × 10^4^ hCPCs were seeded onto each d-HuSk bearing-well and cultured under standard static culture conditions using the same medium. Sterilized and rehydrated d-HuM (*n* = 3) sections were seeded and cultured in the same conditions and used as a reference. Beginning at 48 h after seeding, and then every day for 1 week, cell death rate was assessed using trypan blue exclusion assay following a previously described protocol with some modifications (Di Meglio et al., 2017). Specifically, every day cells were detached from a subset of wells in the multiwell plates by incubation with 0.25% trypsin-EDTA solution (Sigma-Aldrich) for 10 min. Detached cells were then stained with trypan blue stain (0.4% in PBS) (Lonza, Walkersville, MD, United States) for 2 min at room temperature and counted by three independent observers using a hemocytometer. Due to a damage of the plasma membrane, dead cells uptake the dye but are not capable of excluding it and stain blue. Alive cells, instead, exclude the dye and can be recognized among the dead blue cells as unstained cells. Therefore, such assay allows the computation of both cell death rate and cell viability. The percentage of dead cells and of alive cells over total cells for each time point was calculated and expressed as the mean percentages of the total number of cells ± SEM.

### Scanning Electron Microscopy

Six hundred-micrometer -thick cryosections of d-HuSk placed in 35-mm culture dishes were sterilized, rehydrated and repopulated with 5 × 10^5^ hCPCs as described above and cultured under standard culture conditions for 4 weeks. Surface ultrastructure was then studied by Scanning Electron Microscopy (SEM). Briefly, samples fixed in 10% neutral-buffered formalin were dehydrated with ascending ethanol series (30–100%), subjected to Critical Point Dryer (EMITECH K850), mounted on a stub and sputtered with platinum-palladium Denton Vacuum (DESK V). FESEM (Field-Emission SEM) Supra 40 (ZEISS; EHT = 5.00 kV, WD = 22 mm, detector in lens) was used for observation.

### Gene Expression Analysis

Total RNA was extracted from hCPCs cultured on d-HuSk (*n* = 4) and on d-HuM (*n* = 4) for 4 weeks. RNA was dissolved in RNase-free water, and the final concentration was determined using a Nanodrop 1000 spectrophotometer (Thermo Scientific). Then, 100 ng of RNA extracted from each sample was reverse transcribed into cDNA using a QuantiTect Reverse Transcription Kit (Qiagen) and gene expression analysis for genes specific for cardiac program, like *GATA4* and *TBX5*, and for cardiac myocytes, like *CX43*, *CX37*, *TBX3*, *TBX5*, *MEF2C*, *ACTC1*, *MYH7* (Supplementary Table S4) was performed by real-time PCR as previously described (Di Meglio et al., 2010). All samples were tested in triplicate with the housekeeping gene (*GAPDH*). Comparative quantification of target gene expression in the samples was performed based on the cycle threshold (Ct) normalized to the housekeeping gene.

### Immunofluorescence

Immunofluorescence experiments were performed on hCPCs cultured on d-HuSk for 4 weeks using primary antibodies specific for actin (α-sarcomeric) (Sigma-Aldrich), Connexin-43 (Abcam, Cambridge, United Kingdom), desmin and dystrophin (Sigma-Aldrich) following a previously published protocol (Castaldo et al., 2013; Nurzynska et al., 2013). Microscopic analyses were performed with a Nikon Eclipse Ti-E Microscope DS-Qi2 by NIS Elements software (Nikon Instruments, Tokyo, Japan). The expression of cardiac myocyte differentiation markers was also analyzed by confocal microscopy. To this aim, formalin-fixed samples were permeabilized for 3 h with a permeabilizing solution containing 1% BSA and 0.1% Triton in PBS, then blocked with 5% donkey serum and incubated overnight with primary antibodies. Then samples were extensively washed with PBS and incubated for 90 min with a fluorescein-conjugated donkey anti-mouse (Jackson ImmunoResearch Europe). Rhodamin-conjugated phalloidin was used to detect F-actin while TO-PRO3 iodide fluorescent dye 642/661 (Invitrogen) was used for nuclear staining. As a negative control, the primary antibody was omitted. Microscopic analysis and digital microphotography was performed by a Leica Confocal Microscope (Laser Scanning TCS SP2 equipped with Kr/Ar and He/Ne lasers) performing optical spatial series with a step size of 2 μm.

### Statistical Analysis

Statistical analysis was performed using GraphPad Prism version 5.00 for Windows (GraphPad Software, San Diego, CA, United States^2^). Data obtained from the growth factor array, trypan blue exclusion assay and qPCR were analyzed using *t*-test, while data obtained from the analyses of Elastin and GAG content were analyzed using one-way analysis of variance (ANOVA) with Tukey’s post-test. Further, two-way ANOVA was adopted to determine how the response to uniaxial tensile tests was affected by the “donor” and the “orientation” factors. All experiments were performed in triplicate and all data were expressed as the mean ± SEM. A value of *p* ≤ 0.05 was used to identify any statistically significant differences.

## Results

### Evaluation of the Decellularization Procedure

**FIGURE 1:**
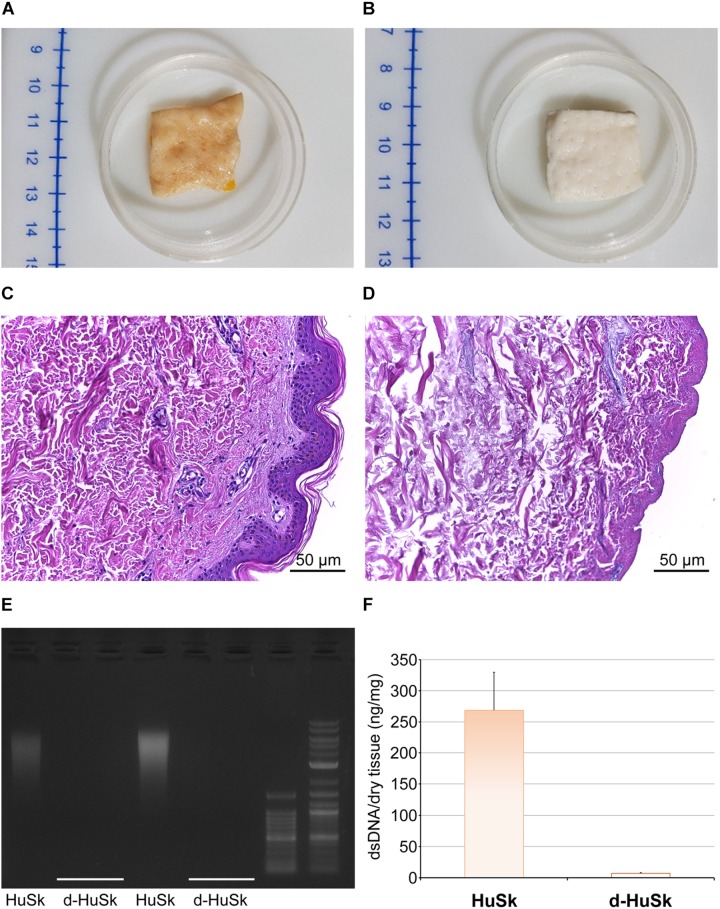
Evaluation of the effectiveness of the decellularization procedure of adult human skin. Representative images of macroscopic examination of native human skin (HuSk) **(A)** and decellularized human skin (d-HuSk) **(B)** showing an obvious change in color after decellularization. Representative images of hematoxylin and eosin staining comparing HuSk **(C)** with d-HuSk **(D)** and showing the preserved tissue architecture and the absence of nuclei in d-HuSk (Scale bar: 50 μm). **(E)** Representative gel electrophoresis revealing an extremely low content of residual dsDNA in d-HuSk when compared with HuSk. **(F)** Quantification of dsDNA in d-HuSk showing a content well below that of HuSk and the proposed criterion of 50 ng.

The first obvious change that occurred with the decellularization was macroscopic, as with respect to the brownish color of native skin (Figure 1A) the d-HuSk samples turned completely white (Figure 1B). Hematoxylin and Eosin staining, and quantitative measurement of DNA content were then used to evaluate the effectiveness of decellularization. When compared with native skin (Figure 1C), the noticeable absence of nuclei in d-HuSk (Figure 1D) emerged from the histological analysis while the residual dsDNA content in d-HuSk, as measured by quantitative analysis, resulted as low as 7.50 ± 2.162 ng per mg of dry tissue (Figures 1E,F). Therefore, d-HuSk fulfilled the requirements proposed for the evaluation of the effectiveness of decellularization procedures, which include the absence of nuclei as revealed by histology and a content of dsDNA per mg of dry tissue below the set threshold of 50 ng (Crapo et al., 2011). An overall well-preserved organization of the dermal connective tissue in d-HuSk was also apparent at the histological analysis (Figure 1D).

### Analysis of Decellularized Dermal Matrix Composition

**FIGURE 2:**
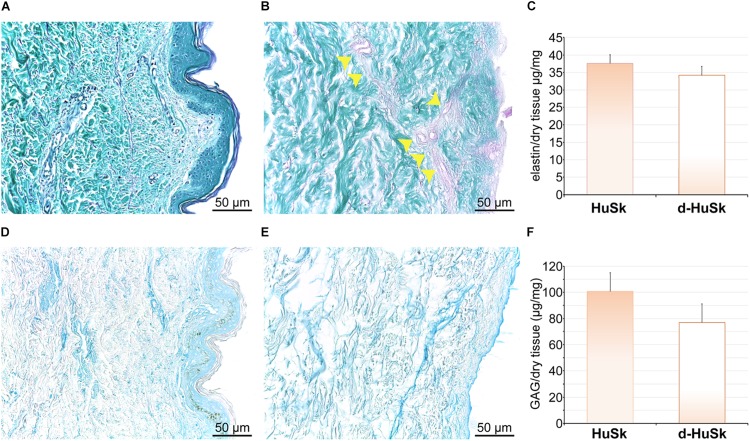
Histochemical and quantitative analysis of elastin and GAG content in native human skin (HuSk) and in d-HuSk. Representative images of the histochemical analysis performed by Paraldehyde fuchsin Gomori staining showing the presence of elastic fibers in HuSk **(A)** and d-HuSk **(B)**. Patent blood vessels surrounded by abundant elastic fibers are apparent in d-HuSk (yellow arrowheads). **(C)** Quantification of elastin by specific dye-binding assay showing comparable content of elastin in d-HuSk and HuSk. Representative images of the histochemical analysis performed by Alcian Blue staining and showing by the light blue color the presence of GAGs in HuSk **(D)** and d-HuSk **(E)**. **(F)** Quantification of GAGs by specific dye-binding assay showing no statistically significant difference in content of GAGs in d-HuSk and HuSk. Scale bar is 50 μm.

**FIGURE 3:**
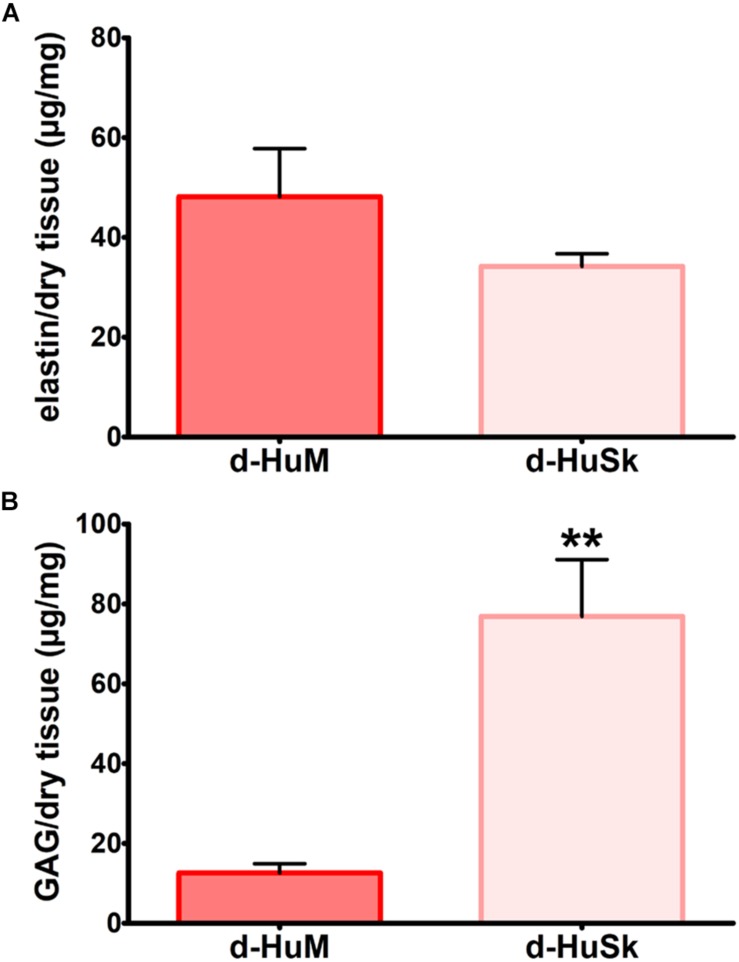
Comparative analysis of the content of elastin and GAGs in decellularized human myocardium (d-HuM) and d-HuSk. Graphs comparing the content of elastin **(A)** and GAG **(B)** between d-HuM and d-HuSk evaluated by specific quantitative dye-binding assays. Double asterisks indicate very significant difference (**p ≤ 0.01).

Paraldehyde fuchsin Gomori staining and Alcian blue staining were used to evaluate the effects of the decellularization procedure on dermal architecture and to assess the retention and distribution of elastic fibers and GAGs in d-HuSk. Additionally, quantitative dye-binding assays were used to perform comparative analyses in elastin and GAG content between d-HuSk and native skin to confirm the effectiveness of decellularization in preserving matrix composition, and between d-HuSk and d-HuM to evaluate whether the content of the investigated components of the ECM in d-HuSk was comparable to that in cardiac matrix. Paraldehyde fuchsin Gomori staining clearly showed the presence of elastic fibers in native skin (Figure 2A) and in d-HuSk (Figure 2B), where the retention and distribution of elastic fibers resulted more obvious in the connective tissue surrounding well-preserved blood vessels (Figure 2B). Quantitative Fastin Elastin assay not only confirmed the presence of elastin in d-HuSk but also showed that elastin content did not differ significantly from that of native skin (34.205 ± 2.529 μg/mg of dry tissue in d-HuSk vs. 37.577 ± 2.561 μg/mg of dry tissue in native skin) (Figure 2C). Similarly, Alcian blue staining revealed the presence of GAGs in native skin (Figure 2D) and their retention in d-HuSk (Figure 2E), while quantitative Blyscan assay provided further confirmation of the effectiveness of decellularization procedure in producing a well-preserved dermal matrix containing amounts of GAGs that did not differ significantly from those of native skin (76.89 ± 14.22 μg/mg of dry tissue in d-HuSk vs. 100.70 ± 17.36 μg/mg of dry tissue in native skin) (Figure 2F). Conversely, while the elastin content of d-HuSk did not differ significantly from that of d-HuM (34.205 ± 2.529 μg/mg of dry tissue vs. 48.18 ± 9.629 μg/mg of dry tissue) (Figure 3A), quantitative analysis revealed in d-HuSk a significantly higher content of GAGs than in d-HuM (76.89 ± 14.220 μg/mg of dry tissue vs. 12.60 ± 2.30 μg/mg of dry tissue, *p* ≤ 0.001) (Figure 3B).

**FIGURE 4:**
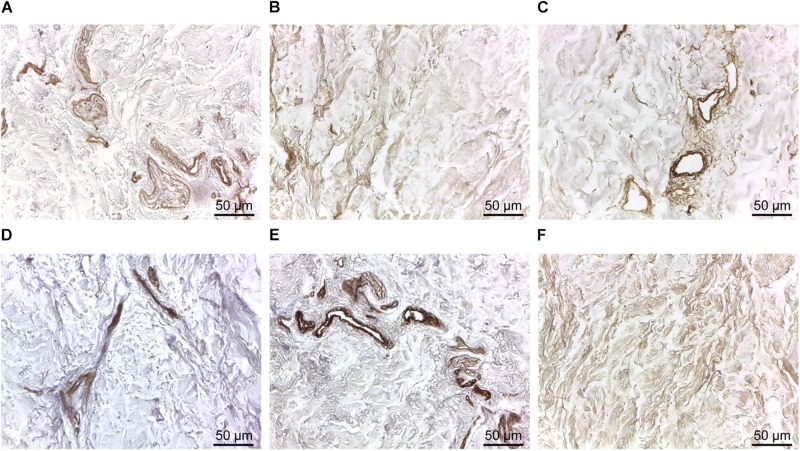
Immunodetection of collagenous and non-collagenous ECM proteins in d-HuSk. Representative images of the immunohistochemical analysis showing the presence and localization in d-HuSk of type I **(A)**, type III **(B)**, and type IV **(C)** collagen, and of fibronectin **(D)**, laminin **(E)**, and tenascin **(F)**. Scale bar is 50 μm.

**FIGURE 5:**
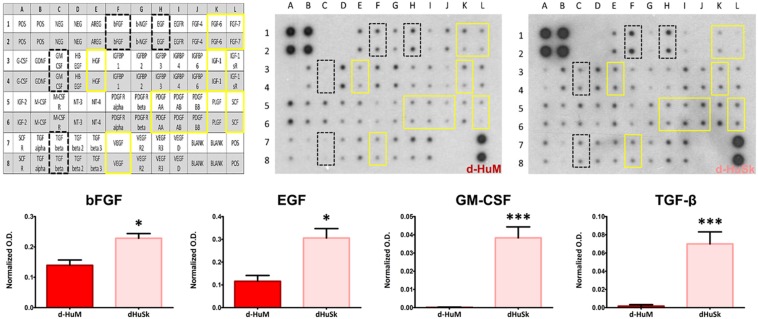
Comparative analysis and quantification of growth factor content in d-HuM and d-HuSk. The representative images of the protein array membranes showed that nine of the targeted growth factors were present both in d-HuM and d-HuSk without any significant difference (yellow solid squares), while bFGF, EGF, GM-CSF, and TGF-beta (black dotted squares) were present at a significantly higher concentration in d-HuSk, as confirmed by the quantification shown by the graphs. In the upper left corner, the array map is shown as a reference. Asterisks were used to report significance in each comparison as follows: significant (*p ≤ 0.05) and very significant (***p ≤ 0.001). O.D.: optical density.

To further evaluate the suitability of d-HuSk as substitute for cardiac ECM, we evaluated by immunohistochemistry the presence and localization in d-HuSk of structural and functional proteins that are components of both cardiac and dermal ECM. Immunohistochemical analysis revealed that d-HuSk contained ECM proteins of critical importance for myocardium development and maintenance like the types I, III, and IV collagens (Figures 4A–C), and the non-collagenous proteins fibronectin, laminin, and tenascin (Figures 4D–F). Types I and III (Figures 4A,B) collagens along with fibronectin and tenascin (Figures 4D,F) were scattered throughout the dermis, where type I collagen (Figure 4A) and fibronectin (Figure 4D) were visible as thicker bundles, while type III collagen (Figure 4B) and tenascin (Figure 4F) formed a delicate texture. Type IV collagen (Figure 4C) and laminin (Figure 4E) were localized mostly in the basement membrane of vessels, instead. Since the ECM is known to function as storage for growth factors, we performed a comparative analysis of the growth factor profile of d-HuSk and d-HuM by protein array (Figure 5). The analysis revealed that the two matrices contained, to a large extent, the same growth factors, including hepatocyte growth factor (HGF), insulin-like growth factor (IGF), stem cell factor (SCF), platelet-derived growth factor (PDGF), and vascular endothelial growth factor (VEGF). Additionally, d-HuSk resulted enriched with growth factors that were virtually absent in d-HuM, like granulocyte-macrophage colony-stimulating factor (GMCSF) and transforming growth factor (TGF-beta), and contained significantly higher amount of growth factors like basic fibroblast growth factor (bFGF) and epidermal growth factor (EGF).

### Mechanical Characterization

**FIGURE 6:**
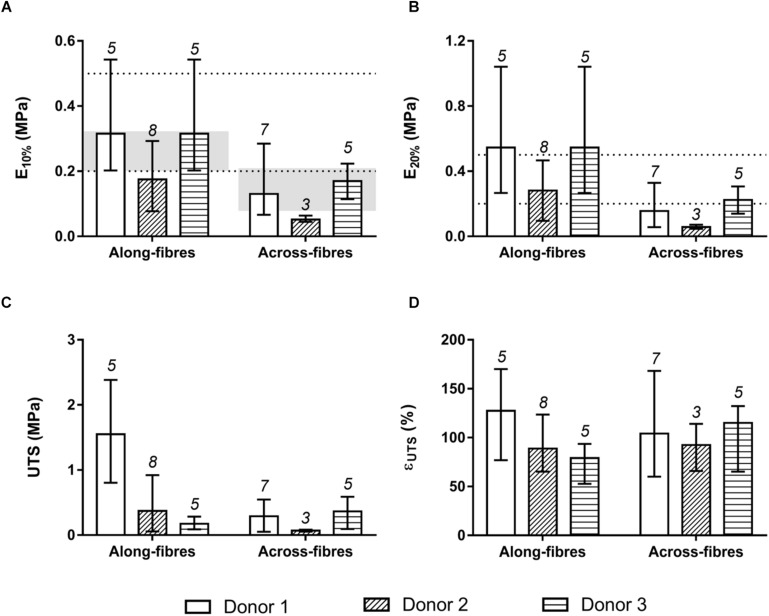
Mechanical characterization by uniaxial tensile stretching of d-HuSk specimens oriented along and across the Langer’s lines. **(A)** Average values and ranges of variation of elastic modulus *E*_10__%_ measured at 10% of strain. The dotted lines delimit the range of elastic modulus values reported in literature for human myocardium at the end of diastole (0.2–0.5 MPa), while the gray-colored bands refer to human myocardium elastic moduli values calculated by Sommer et al. (2015) along and across the fibres. **(B)** Average values and ranges of variation of elastic modulus *E*_20__%_ measured at 20% of strain. **(C)** Average values and ranges of variation of ultimate tensile strength UTS. **(D)** Average values and ranges of variation of ultimate strain ε_*UTS*_.

To investigate the d-HuSk mechanical behavior, uniaxial tensile tests were performed on d-HuSk specimens oriented along and across the Langer’s lines. Focusing on the human left ventricular longitudinal strain range (Kuznetsova et al., 2008), specimens oriented along the Langer’s lines presented, at 10% of strain, average elastic modulus *E*_10__%_ values equal to 0.25 ± 0.13 MPa, and, at 20% of strain, average elastic modulus *E*_20__%_ values equal to 0.42 ± 0.26 MPa (Figures 6A,B). These values were comparable both with that reported in literature for native human myocardium at the end of diastole (0.2–0.5 MPa, dotted lines in Figures 6A,B) (Jawad et al., 2007; Chen et al., 2008; Bouten et al., 2011), and with human myocardium elastic moduli values measured by Sommer et al. (2015) along (0.26 ± 0.06 MPa) and across (0.15 ± 0.06 MPa) the fibers, calculated as the slope of the mean equi-biaxial engineering stress-strain curves at 10% strain on human myocardial tissue samples from 26 subjects (gray-colored bands in Figure 6A, respectively). Measured *E*_10__%_ and *E*_20__%_ values of specimens oriented along the Langer’s lines were higher (on average: +58.4% ± 12.1% for *E*_10__%_; +69.9% ± 10.3% for *E*_20__%_; *p* < 0.0001) than the average elastic moduli measured for the across-fiber specimens (0.12 ± 0.07 MPa for *E*_10__%_, Figure 6A; 0.15 ± 0.09 MPa for *E*_20__%_, Figure 6B). Moreover, specimens oriented along Langer’s lines showed a higher rupture resistance in terms of ultimate tensile strength UTS (Figure 6C), although the ultimate strain ε_*UTS*_ values did not present significant differences (Figure 6D). The only exception was represented by specimens from the third donor, characterized by higher UTS values in across-fiber specimens, however, this discrepancy was not statistically significant. Variance analyses are reported in Supplementary Tables S2, S3. Both the “donor” and the “orientation” factors were significant when considered individually (*p* < 0.05), with the only exception of ε_*UTS*_ which did not show significant differences.

### Effects of Decellularized Dermal Matrix on hCPCs *in vitro*

**FIGURE 7:**
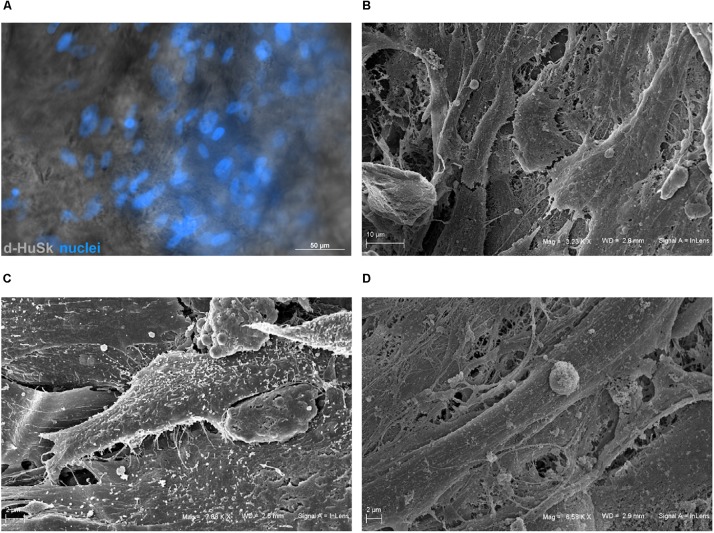
Microscopic analysis of hCPCs engraftment on d-HuSk. **(A)** Representative image of fluorescence and phase contrast microscope analysis showing by the nuclear staining with DAPI the presence of hCPCs engrafted onto d-HuSk. Representative images of SEM analysis showing two hCPCs that contact each other **(B)**, one cell with elongated irregular shape characterized by multiple filopodia **(C)** and a cell with an elongated rectangular shape **(D)**. [Scale bar: 50 μm for **(A)**, 10 μm for **(B)**, and 2 μm for **(C,D)**].

To evaluate the ability of d-HuSk to serve as a viable substitute for cardiac native microenvironment, we seeded and cultured hCPCs on d-HuSk and used as a reference hCPCs seeded and cultured on d-HuM in the same conditions. By binding the nuclear DNA, the DAPI staining showed the presence of hCPCs that engrafted onto d-HuSk (Figure 7A), while SEM analysis not only confirmed the engraftment of hCPCs on d-HuSk but also allowed to visualize the cell-to-cell contacts (Figure 7B) and to examine the cellular morphology. hCPCs engrafted on d-ECM appeared either as mesenchymal-like cells characterized by elongated irregular shape and multiple filopodia (Figure 7C) or as rectangular/polygonal-shaped cells resembling differentiating cardiomyocyte (Figure 7D).

**FIGURE 8:**
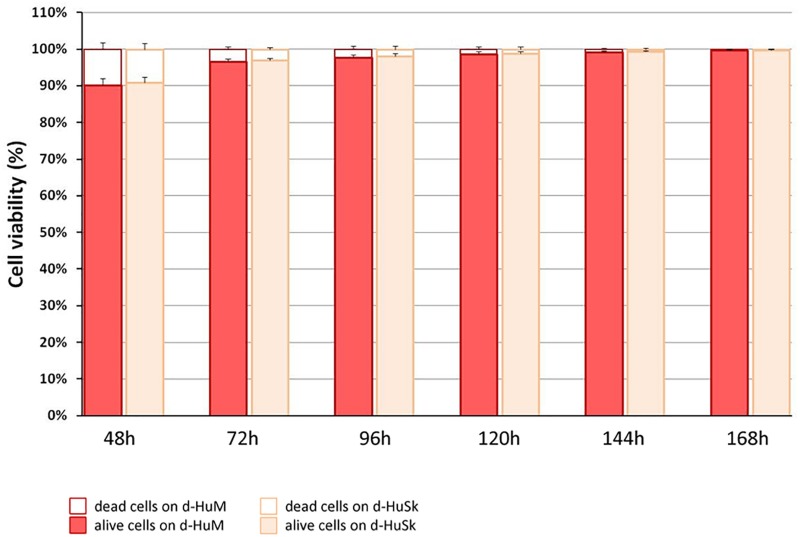
Quantification of cell death rate and viability of hCPCs on d-HuM and d-HuSk. Mean death rate and mean viability of hCPCs cultured on d-HuSk or d-HuM, as measured by trypan blue exclusion assay. With time cell death rate dramatically decreased from around 9% to less than 1%, without any statistically significant differences between cells cultured on d-HuM or d-HuSk.

**FIGURE 9:**
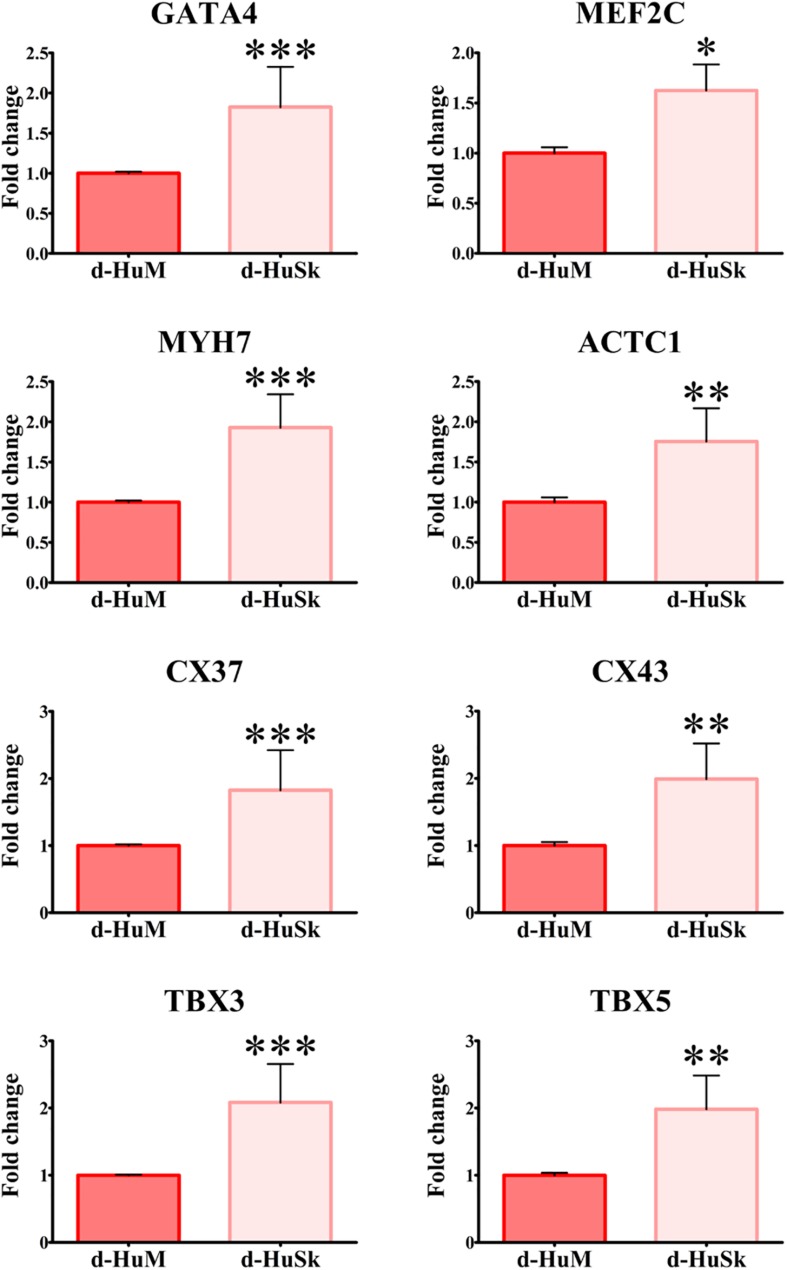
Gene expression analysis of cardiac myocyte markers in hCPCs cultured on d-HuM and d-HuSk for 4 weeks. Real-time PCR analysis of the expression of genes characteristic of cardiac myocytes showing an upregulation of the transcription for all markers in hCPCs cultured on d-HuSk when compared with hCPCs cultured on d-HuM. Asterisks are indicators of the p value obtained in each comparison as follows: significant (*p ≤ 0.05), very significant (**p ≤ 0.01), and extremely significant (***p ≤ 0.001).

**FIGURE 10:**
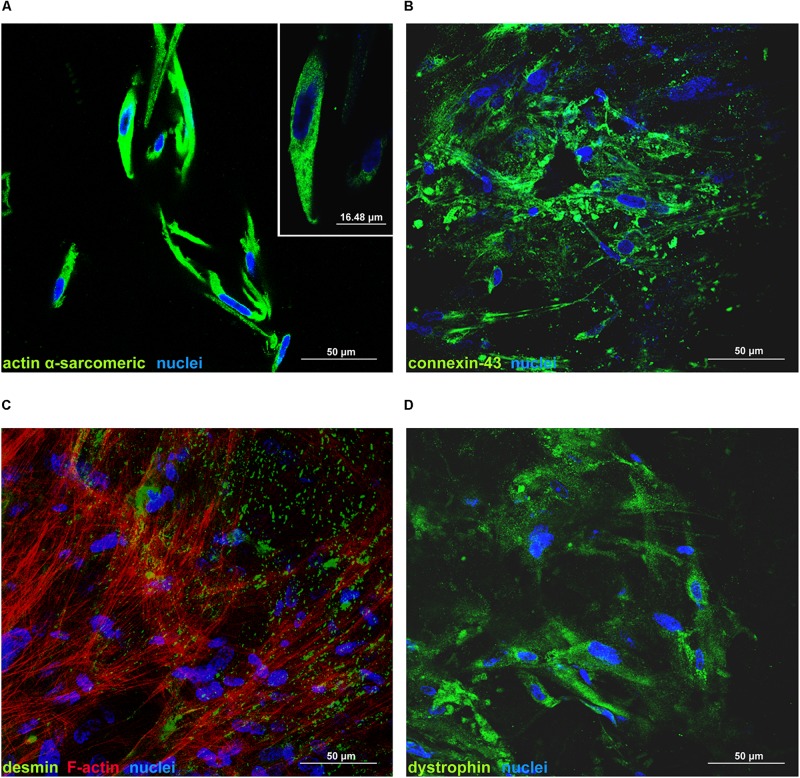
Immunofluorescence analysis of the expression of cardiac myocyte markers by hCPCs cultured on d-HuSk for 4 weeks. Representative images of confocal microscopy analyses showing the immunopositivity and distribution of alpha-sarcomeric actin **(A)**, connexin-43 **(B)**, desmin along with F-actin **(C)**, and dystrophin **(D)** in hCPCs cultured on d-HuSk. The inset in **(A)** shows at higher magnification the expression of alpha-sarcomeric actin. (Scale bar: 50 μm in all pictures, 16.48 μm in the inset).

Cell death rate and cell viability of hCPCs cultured on d-HuSK or on d-HuM were quantified using a trypan blue exclusion assay. The death rate of hCPCs measured 48 h after seeding did not differ significantly between d-HuM and d-HuSk (9.814 ± 1.792% and 9.112 ± 1.532%, respectively) and on both matrices the hCPC death rate dramatically decreased with time, till it reached values well below 1% of total cells, without any statistically significant differences between the two matrices (0.257 ± 0.107% on d-HuM and 0.253 ± 0.104% on d-HuSk). Obviously, cell viability had an inverted trend and increased with time on both d-HuM and d-HuSk. Specifically, the mean percentage of live cells was 90.186 ± 1.792% and 90.888 ± 1.792% after 48 h on d-HuM and d-HuSk, respectively, but increased up to 99.743 ± 0.107% and 99.747 ± 0.107% after 7 days on d-HuM and d-HuSk, respectively (Figure 8). Finally, to assess the suitability of d-HuSk to serve as a cardiogenic environment for hCPCs, we analyzed the expression of cardiac myocyte differentiation markers, again using as a reference the expression of the same markers in hCPCs cultured in the same conditions on d-HuM. Gene expression analysis showed that not only hCPCs cultured on d-HuSk for 4 weeks retained the expression of markers typical of cardiac myocytes, but the transcription of all investigated genes was also significantly up-regulated (*p* ≤ 0.05) up to twofold in hCPCs cultured on d-HuSk (Figure 9). Moreover, the expression of cardiac myocyte specific markers by hCPCs engrafted onto d-HuSk was further evaluated by immunocytochemistry and the analyses at the fluorescence or confocal microscope showed the immunopositivity of hCPCs engrafted onto d-HuSk for the markers investigated. Clearly, the main cell population of hCPCs consisted of cells expressing alpha-sarcomeric actin and connexin-43 (Figures 10A,B), as well as desmin and dystrophin (Figures 10C,D) that are all markers of cardiac myocytes. The confocal microscope revealed as F-actin filaments distributed in approximately parallel rows, a pattern that suggests cardiomyocyte differentiation.

## Discussion

Projections show that by 2030 the prevalence of IHD will increase about 18% from 2013 estimates and almost 23.6 million people will die from CVDs, mainly from heart disease and stroke (Mozaffarian et al., 2016). Although not all coronary events are lethal, most of them lead to the formation of a permanent scar that, rather than merely be an inert region of the heart wall, holds the potential to complicate the clinical course by promoting reactive fibrosis in the uninjured myocardium (Schelbert et al., 2014). Indeed, reparative scar tissue, through pro-fibrotic signaling factors that induce transdifferentiation of fibroblast to myofibroblasts and ECM deposition (Frangogiannis, 2014; Talman and Ruskoaho, 2016), might eventually lead to heart failure. No current pharmacological therapy holds the potential to restore the histological integrity of the myocardium. Therefore, engineered cardiac tissue, developed through a combination of supporting scaffolds, cardiac specific cell lineages with regenerative ability, biological signals, such as growth factors and ECM components, and mechanical and electrical stimulation (Zimmermann et al., 2000; Radisic et al., 2004; Zhang et al., 2013; Tiburcy et al., 2017; Ronaldson-Bouchard et al., 2018), is rapidly and powerfully emerging as the only alternative therapeutic approach with the potential to accomplish the challenging task of reestablishing the structural, biomechanical and functional integrity of ischemic myocardium. Unquestionably, the best-performing scaffold in terms of both biological signaling and compliance with cardiomyocyte contraction is the native cardiac ECM. However, even though such simple and obvious consideration has triggered in the last decade a multitude of studies aimed at obtaining biological scaffolds for CTE from native matrix through the decellularization of the myocardium (Moroni and Mirabella, 2014; Oberwallner et al., 2014), the dramatically unbalanced ratio between demand and availability of healthy human hearts has forced the search for alternative scaffolds. Needless to say, the ideal scaffold should recapitulate the composition and the mechanical properties of the native cardiac environment (Ye and Black, 2011). Additionally, biocompatibility and biodegradability are key requirements needed for a scaffold to be considered of clinical value and, not of secondary importance, any scaffold should be cost effective to become commercially viable (O’Brien, 2011). Most of the aforementioned requisites needed for determining the suitability of scaffolds for CTE might be possessed by biological scaffolds produced by decellularization of tissues. However, to serve as substitute for the cardiac ECM the tissue/organ should have similarities with the cardiac environment and the decellularization process produce a thoroughly acellular scaffold without disrupting the ECM composition. Therefore, we explored the possibility of using d-HuSk as an autologous, cost effective and easily accessible scaffold for CTE and evaluated *in vitro* its capability to support resident CPC engraftment, survival and differentiation potential.

Following a recently described protocol for the decellularization of the adult human heart (Di Meglio et al., 2017), the adult human skin was easily and successfully decellularized to produce a scaffold that, being deprived of any nuclear remnants and residual dsDNA (Figure 1), met the criteria proposed to satisfy the intent of decellularization (Crapo et al., 2011). Nevertheless, although skin matrix consists of proteins that are also found in the cardiac matrix (Uitto et al., 1989; Lockhart et al., 2011) and is biocompatible by definition, thorough removal of resident cells is a necessary but not sufficient criterion to ensure safety and grant clinical use of d-HuSk. Indeed, the treatment with decellularizing detergents could cause biological impoverishment of the ECM, by disrupting crucial proteins of cardiac interstitium and removing soluble factors, and leave potentially harmful compounds that might impair scaffold recellularization and prevent its clinical use (Kawecki et al., 2018). Importantly, other than providing structural integrity and connecting muscle cells and blood vessels, the interstitial connective tissue of the myocardium is known to provide biological and mechanical cues that are responsible for directing stem cell fate (Ahmed and Ffrench-Constant, 2016) and cell behavior during cardiac development, adult myocardium maintenance and physiological response to pathological stimuli (Guilak et al., 2009; Gattazzo et al., 2014; Nakayama et al., 2014; Williams and Black, 2015). The investigated ECM components, namely collagens, elastin, GAGs, fibronectin, laminin, and tenascin, are the main structural and functional proteins of cardiac interstitial matrix (Li et al., 2018). The retention of these components in d-HuSk, as proved by histological and quantitative analyses (Figures 2–4), provides evidence to substantiate the ability of d-HuSk to deliver a combination of structural support and biological signals that act in cardiac microenvironment. Specifically, the fibrillar collagens types I and III are the major ECM components of the cardiac embryonic and mature connective tissue network and, together with elastin that is the dominant mammalian elastic protein of the ECM and the main component of elastic fibers (Green et al., 2014), are responsible for conferring tensile strength, resilience and elasticity to the cardiac matrix (Borg et al., 1996). Additionally, they ensure proper myocyte alignment, acting as an adhesive substrate for adjoining myocytes in both developing and adult myocardium (Weber, 1989). Due to their significant water-binding capacity, also GAGs contribute to mechanical stabilization of tissues (Nimeskern et al., 2016) and, as major constituents of cardiac jelly, are implicated in the acquisition of trabeculated wall architecture and septation during cardiac development (Camenisch et al., 2000, 2002). Cardiac jelly is the primitive cardiac ECM that mechanically connects the inner endocardial layer and the outer myocardium layer of the tubular heart and that forms a thick viscoelastic layer that mechanically supports the valveless pumping function of the embryonic heart (Garita et al., 2011). GAGs control the hydration of the cardiac jelly generating turgor pressure that is involved in the remodeling process of the ventricular tube that initiates trabeculation and formation of cardiac chambers (Farouz et al., 2015; Männer and Yelbuz, 2019). Further important components of d-HuSk that are also crucial components of the cardiac matrix, since its earliest form as cardiac jelly, include the non-collagenous glycoproteins fibronectin and laminin. Fibronectin is a fibrillar protein whose significant influence over mechanical properties of the matrix and behavior of adhering cells has been extensively documented in embryonic and adult heart. The profound implication of fibronectin in mesodermal migration, adhesion and differentiation as well as in embryonic cardiomyocyte proliferation is testified by the cardiovascular defects that develop at later stages of embryogenesis in fibronectin deficient embryos and that are incompatible with life (Little and Rongish, 1995; Rienks et al., 2014; Williams and Black, 2015). Although fibronectin decreases with developmental age, it is still required in the adult heart for providing structural support by linking the cardiac interstitium with the basement membranes of endothelial and muscle cells and for the reparative response following a myocardial infarction (Borg et al., 1984; Kim et al., 1999; Konstandin et al., 2013). Conversely, laminin expression is constant throughout life and, while heart organogenesis does not proceed in the absence of laminin, in the post-natal heart it is a major component of the basement membrane that surrounds mature cardiomyocytes and acts as an adhesive ligand (Castaldo et al., 2008). Furthermore, tenascin C is a matricellular protein highly but transiently expressed during the embryonic development of the heart that reappears in various pathological conditions. Its spatiotemporally restricted expression is critical to promote the differentiation of cardiomyocytes in the embryonic heart (Imanaka-Yoshida et al., 2003) and the recruitment of myofibroblasts in tissue remodeling during several heart diseases like myocardial infarction (Imanaka-Yoshida et al., 2014). It has also been reported that tenascin C is an elastic molecule that can be stretched to several times its resting length *in vitro* (Oberhauser et al., 1998; Marín et al., 2003) and contributes to tissue elasticity (Imanaka-Yoshida and Aoki, 2014). Additionally, other than holding the potential to provide cardiac-like biochemical cues, d-HuSk also has the added value of hosting very well preserved vessels, as shown by the histological analysis (Figure 2). Although its patency has yet to be verified, this residual vascular network can play a major role in the diffusion of nutrients that supports cell survival and differentiation.

However, along with the cardiac-like biochemical and structural behavior, the ideal scaffold for CTE should also recapitulate the resultant mechanical properties of the human healthy myocardium. Healthy myocardium is characterized by an anisotropic hierarchical structure and a non-linear viscoelastic mechanical behavior (Pislaru et al., 2014), for which both the cardiac matrix and mostly the cellular component are responsible. Elasticity, indeed, is an intrinsic property of cardiomyocytes largely conferred by sarcomere structure and critical for the contraction and relaxation during cardiac cycle (Janssen, 2010; Ait Mou et al., 2015). Obviously, muscle cells need a compliant matrix able to elastically deform during contraction-relaxation cycles (Hanson et al., 2013). Similarly, human skin reacts to loadings with a viscoelastic, anisotropic dual behavior, and it is characterized by a preferential collagen fiber orientation, referred to as Langer’s lines (Langer, 1978). At low strain values, elastin fibers are responsible for skin mechanical behavior, whereas at large strains collagen fibers become dominant (Terzini et al., 2016). To investigate the mechanical performance of d-HuSk, we performed uniaxial tensile tests on d-HuSk specimens oriented along and across the Langer’s lines. Applying a strain range typical of the human left ventricle (Kuznetsova et al., 2008), d-HuSk specimens oriented along the Langer’s lines presented average elastic moduli *E*_10__%_ and *E*_20__%_ comparable with literature data on native human myocardium (Jawad et al., 2007; Chen et al., 2008; Bouten et al., 2011; Sommer et al., 2015), and higher than elastic moduli values measured for the across-fiber specimens (Figure 6). Mechanical characterization of d-HuSk confirms that skin elasticity is mostly provided by the ECM rather than by resident cells, and the d-HuSk elasticity guarantees a mechanical behavior suitable for CTE application. Moreover, d-HuSk presents directionally dependent anisotropic behavior depending on the ECM protein fiber network predominantly oriented along the Langer’s lines (Demer and Yin, 1983) and similar with the structural organization of the myocardium (Gilbert et al., 2007).

Based on this intriguing resemblance between d-HuSk and cardiac ECM, it is tempting to speculate that d-HuSk might function as a scaffold to support and boost cardiac differentiation in a stem/progenitor cell-based cardiac regeneration approach. Nonetheless, to be considered suitable for regenerative medicine purposes, any scaffold must first be biocompatible (O’Brien, 2011). This implies that it must have the ability to attract cells and provide a comfortable environment capable to support or promote cell adhesion and survival. Tests of *in vitro* recellularization of d-HuSk with hCPCs proved that d-HuSk provided an environment as effective and safe as the native cardiac matrix in that it supported engraftment (Figure 7) and survival (Figure 8) of hCPCs. Additionally, the positive effect of a biological scaffold loaded with signals that direct and control cardiac development and homeostasis on the expression of markers specific for cardiomyocytes by hCPCs was somehow predictable. It was yet astounding that the expression of markers specific for cardiomyocytes in hCPCs cultured on d-HuSk not only persisted, but resulted also up-regulated, when compared with that of the same cell population cultured on d-HuM in the same conditions (Figures 9, 10). Such evidence, along with the morphology of cells observed at the SEM (Figure 7), supports the hypothesis that d-HuSk might constitute a myocardial-like environment that ensures the survival and sustains the differentiation of resident CPCs. Accordingly, when compared with d-HuM, d-HuSk also contained growth factors that are commonly stored in the native cardiac matrix, like HGF, IGF, SCF, PDGF, and VEGF, and was enriched with growth factors like bFGF, EGF, GM-CSF, and TGF-beta (Figure 5). These factors are involved in a variety of cardiac processes like cardiac development (Saetrum Opgaard and Wang, 2005; Deshwar et al., 2016), mobilization and proliferation of endothelial progenitor cells (Qiu et al., 2014), angiogenesis and neovascularization (Murakami and Simons, 2008; Beohar et al., 2010), cardiomyocyte proliferation and differentiation (Ma et al., 2017; Farzaneh et al., 2019), cardioprotection (Zhu et al., 2017), cardiac remodeling (Sala and Crepaldi, 2011), and repair (Vandervelde et al., 2005). Such abundance of growth factors in d-HuSk strengthens its suitability for CTE as a promising tool capable of providing alone two of the three pillars of tissue engineering (O’Brien, 2011), namely the scaffold and the signals, and whose potential to boost cardiac regeneration as stand-alone or cellularized scaffold is worth being further explored.

The presented work is affected by some limitations. Firstly, the biocompatibility of d-HuSk was evaluated exclusively *in vitro* and in static conditions. Nonetheless, the evidence that CPCs engrafted on a novel and more easily accessible biological scaffold of dermal ECM and retained their differentiation potential represents an important advance in CTE. Indeed, the use of d-HuSk overcomes problems related to the preparation of myocardial biological scaffolds and paves the way for *in vivo* studies that will provide more extensive insights into the clinical utility of d-HuSk. Moreover, to investigate the influence of cardiac-like cyclic stretch stimulation on the matrix and the developing construct, *in vitro* studies within a customized mechanical stretching bioreactor (Putame et al., 2019) are planned.

## Conclusion

We developed a novel biological scaffold for cardiac regenerative medicine that we termed d-HuSk. Obtained from the adult human skin through a simple and effective decellularization procedure (Di Meglio et al., 2017), d-HuSk not only fulfilled the key requirements proposed for the evaluation of the effectiveness of decellularization, but also preserved the histological organization and composition of the native tissue, providing compelling evidence of a highly efficient decellularization method. Other than providing cell attachment sites ensuring cell adhesion, d-HuSk also retained well-preserved vessels that are critical to ensure diffusion of nutrients, thus addressing two serious problems that frequently affect the use of synthetic biomaterials. Furthermore, the biological nature of d-HuSk ensures a full biodegradability, making d-HuSk a powerful biomaterial. Indeed, a scaffold should be considered a temporary implant that provides support to cells that are, then, expected to replace it with a newly synthesized cardiac matrix. Based on evidence emerging from this work, d-HuSk is a myocardial matrix substitute characterized by myocardium-like biological and mechanical behavior that, despite its different anatomical site of origin, proved to be a suitable environment to support the engraftment, proliferation and differentiation potential of hCPC *in vitro*. If proven *in vivo*, such evidence could have a tremendous potential, as d-HuSk might be used as an autologous scaffold to promote and sustain cardiac regeneration by stem/progenitor cells. Finally, easy accessibility, cost-effective production and autologous origin are all additional groundbreaking features of d-HuSk. In fact, using d-HuSk as an auto-graft would imply transplanting a fully compatible scaffold capable of averting the immunological response and the risk of rejection that affect the heterologous and xenogeneic transplant or implant. Noticeably yet, it has been recently demonstrated that adult fibroblasts isolated from the abdominal skin can be reprogrammed with higher efficiency (Sacco et al., 2019) and that skin fibroblasts can be directly reprogrammed to cardiac mature cells (Cao et al., 2016). Therefore, the preparation of d-HuSk scaffolds holds the potential to eventually evolve into the development of a more complex therapeutic strategy aimed at constructing, with one single intervention of skin harvesting, a fully autologous engineered myocardium using cardiac direct reprogramming of fibroblast to restore the cellular compartment and the decellularized dermal ECM to replace the extracellular compartment.

## Data Availability Statement

All data supporting the findings of this study are available within the article and its Supplementary Information; source data for the figures in this study are available from the authors upon request.

## Ethics Statement

This study was carried out following protocols approved by the Hospital Ethical Committee. Patients provided written informed consent in conformity with the principles outlined in the Declaration of Helsinki.

## Author Contributions

IB, VR, AS, GR, DM, SM, FD, and CC contributed conception and design of the study. IB, VR, AS, GR, MC, AC, CS, DN, FSi, FD, and CC conducted experiments and analyzed experimental data. DM, MT, AA, and GS performed mechanical characterization and analysis. CC wrote the first draft of the manuscript. GR, MC, DM, and MT wrote sections of the manuscript. FSc and FD’A provided the surgical specimens. All authors contributed to manuscript revision, read and approved the submitted version. IB, VR, and AS are equal contributors.

## Conflict of Interest

The authors declare that the research was conducted in the absence of any commercial or financial relationships that could be construed as a potential conflict of interest.

## Footnotes

1 https://imagej.nih.gov/ij/download.html

2 www.graphpad.com
