# Supplementary material for: Decellularized Human Dermal Matrix as a Biological Scaffold for Cardiac Repair and Regeneration
Source: Front Bioeng Biotechnol. 2020 Mar 20;8:229. doi: 10.3389/fbioe.2020.00229 (PMC7099865; doi:10.3389/fbioe.2020.00229)
Supplement: Supplementary file 1 [file Data_Sheet_1.DOCX]

Supplementary Material

# Mechanical parameters extraction

In Supplementary Figure 1-A a representative stress-strain curve for d-HuSk is reported. Highlighted in the figure are the ultimate tensile strength (UTS), evaluated as the maximum value reached by the engineering stress-strain curve, and the ultimate strain (ε_UTS_) evaluated as the strain at which UTS occurs. The dotted square encloses the region of interest for the elastic moduli E_10%_ and E_20%_ extraction. The same region of interest is zoomed in Supplementary Figure 1-B, which contains the representation of the curve slope at 10% and 20% of strain, where the two elastic moduli were computed. The results extracted from the aforementioned method are listed in the Supplementary Table 1.

**
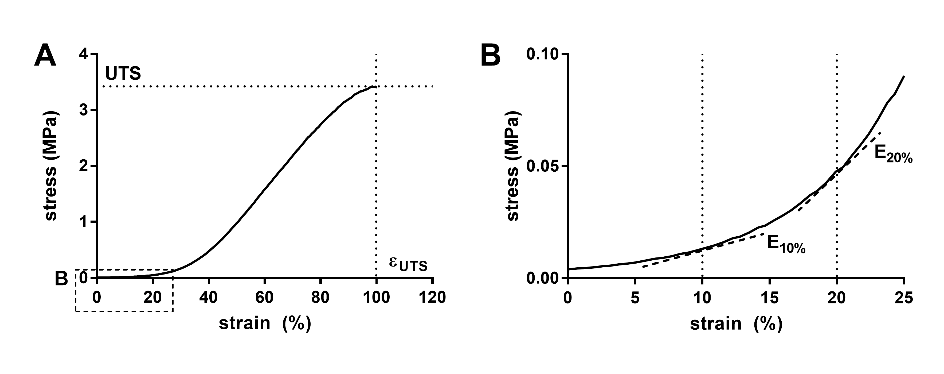
**

**Supplementary Figure 1.** (A) Representative engineering stress-strain curve showing the UTS point and the ε_UTS_; (B) The dotted square encloses the region of interest for E_10%_ and E_20%_ elastic moduli extraction.

## Statistical analysis of mechanical parameters

d-HuSk mechanical properties were reported in relation to the orientation (along and across Langer’s lines) and the donor (Donor 1, Donor 2 and Donor 3). The statistical analysis of the parameters was performed using the two way analysis of variance with a significance level set at p < 0.05. Results are reported in Supplementary Table 2 and 3.
