# Supplementary material for: Decellularized Human Dermal Matrix as a Biological Scaffold for Cardiac Repair and Regeneration
Source: Front Bioeng Biotechnol. 2020 Mar 20;8:229. doi: 10.3389/fbioe.2020.00229 (PMC7099865; doi:10.3389/fbioe.2020.00229)
Supplement: Supplementary file 2 [file Table_1.DOCX]

Supplementary Material

**Supplementary Table 1:** E_10%_, E_20%_, UTS and ε_UTS_ along and across-fibres for the three donors

|  |  | **Donor 1** | | | **Donor 2** | | | **Donor 3** | | |
| --- | --- | --- | --- | --- | --- | --- | --- | --- | --- | --- |
|  |  | **average** | **min** | **max** | **average** | **min** | **Max** | **average** | **min** | **max** |
| E_10%_ (MPa) | Along-fibres | 0.32 | 0.20 | 0.54 | 0.17 | 0.08 | 0.29 | 0.32 | 0.20 | 0.54 |
|  | Across-fibres | 0.13 | 0.07 | 0.28 | 0.05 | 0.04 | 0.06 | 0.17 | 0.11 | 0.22 |
| E_20%_ (MPa) | Along-fibres | 0.55 | 0.27 | 1.04 | 0.28 | 0.10 | 0.47 | 0.55 | 0.27 | 1.04 |
|  | Across-fibres | 0.16 | 0.06 | 0.33 | 0.06 | 0.05 | 0.07 | 0.22 | 0.14 | 0.31 |
| UTS (MPa) | Along-fibres | 1.55 | 0.80 | 2.38 | 0.37 | 0.06 | 0.92 | 0.17 | 0.09 | 0.28 |
|  | Across-fibres | 0.29 | 0.05 | 0.55 | 0.07 | 0.05 | 0.08 | 0.36 | 0.09 | 0.59 |
| ε_UTS_ (%) | Along-fibres | 127.40 | 76.84 | 170.00 | 88.78 | 65.32 | 123.58 | 79.00 | 52.74 | 93.60 |
|  | Across-fibres | 104.07 | 60.16 | 168.30 | 92.46 | 65.96 | 114.02 | 115.12 | 65.28 | 132.22 |
