# Supplementary material for: Decellularized Human Dermal Matrix as a Biological Scaffold for Cardiac Repair and Regeneration
Source: Front Bioeng Biotechnol. 2020 Mar 20;8:229. doi: 10.3389/fbioe.2020.00229 (PMC7099865; doi:10.3389/fbioe.2020.00229)
Supplement: Supplementary file 3 [file Table_2.DOCX]

Supplementary Material

**Supplementary Table 2.** Variance analysis for E_10%_ and E_20%_. Italic characters are used to highlight the not significant factors (p > 0.05).

| **Source** | **Sum Sq.** | **DOF** | **Mean Sq.** | **F** | **p** |
| --- | --- | --- | --- | --- | --- |
| **E_10%_** |  |  |  |  |  |
| Orientation*Donor | 0.01 | 2 | 0.00 | F (2, 27) = 0.2883 | *0.7518* |
| Orientation | 0.17 | 1 | 0.17 | F (1, 27) = 19.6 | 0.0001 |
| Donor | 0.09 | 2 | 0.04 | F (2, 27) = 5.147 | 0.0128 |
| Error | 0.24 | 27 | 0.01 |  |  |
| **E_20%_** |  |  |  |  |  |
| Orientation*Donor | 0.03 | 2 | 0.02 | F (2, 27) = 0.4939 | *0.6157* |
| Orientation | 0.73 | 1 | 0.73 | F (1, 27) = 21.05 | <0.0001 |
| Donor | 0.25 | 2 | 0.12 | F (2, 27) = 3.554 | 0.0426 |
| Error | 0.93 | 27 | 0.03 |  |  |
