# Supplementary material for: Decellularized Human Dermal Matrix as a Biological Scaffold for Cardiac Repair and Regeneration
Source: Front Bioeng Biotechnol. 2020 Mar 20;8:229. doi: 10.3389/fbioe.2020.00229 (PMC7099865; doi:10.3389/fbioe.2020.00229)
Supplement: Supplementary file 4 [file Table_3.DOCX]

Supplementary Material

**Supplementary Table 3.** Variance analysis for UTS and ε_UTS_. Italic characters are used to highlight the not significant factors (p > 0.05).

| **Source** | **Sum Sq.** | **DOF** | **Mean Sq.** | **F** | **p** |
| --- | --- | --- | --- | --- | --- |
| **UTS** |  |  |  |  |  |
| Orientation*Donor | 2.96 | 2 | 1.48 | F (2, 27) = 16.96 | <0.0001 |
| Orientation | 1.58 | 1 | 1.58 | F (1, 27) = 18.05 | 0.0002 |
| Donor | 3.26 | 2 | 1.63 | F (2, 27) = 18.67 | <0.0001 |
| Error | 2.36 | 27 | 0.09 |  |  |
| **ε_UTS_** |  |  |  |  |  |
| Orientation*Donor | 4757.00 | 2 | 2379.00 | F (2, 27) = 2.589 | *0.0936* |
| Orientation | 225.90 | 1 | 225.90 | F (1, 27) = 0.2459 | *0.624* |
| Donor | 3572.00 | 2 | 1786.00 | F (2, 27) = 1.944 | *0.1626* |
| Error | 24803.00 | 27 | 918.60 |  |  |
