# Supplementary material for: Decellularized Human Dermal Matrix as a Biological Scaffold for Cardiac Repair and Regeneration
Source: Front Bioeng Biotechnol. 2020 Mar 20;8:229. doi: 10.3389/fbioe.2020.00229 (PMC7099865; doi:10.3389/fbioe.2020.00229)
Supplement: Supplementary file 5 [file Table_4.DOCX]

Supplementary Material

**Supplementary Table 4:** Primers of genes analyzed by real-time PCR.

| **Gene ID**  **(gene description)** | **Forward sequence**  **Reverse sequence** | **Amplicon length (nt)** |
| --- | --- | --- |
| GAPDH  (glyceraldehyde-3-phosphate dehydrogenase) | 5’-CTCTCTGCTCCTCCTGTTCG-3’  5’-ACGACCAAATCCGTTGACTC-3’ | 114 |
| ACTC1  (actin alpha cardiac muscle 1) | 5’-TCGGGACCTCACTGACTACC-3’  5’-CAAAATCCAGGGCGACATAG-3’ | 125 |
| CX37  (gap junction protein alpha 4) | 5’-CTGATGGGCACCTATGTCG-3’  5’-TCGCTGGCACACAAACAC-3’ | 117 |
| CX43  (gap junction protein alpha 1) | 5’-CGAGGACTATGAGGGCAAGA-3’  5’-TTCAGGTGTGTCGTTGGAAG-3’ | 112 |
| GATA4  (GATA binding protein 4) | 5’-TCATCTCACTACGGGCACAG-3’  5’-GAGGACAGGGTGGATGGAG-3’ | 90 |
| MEF2C  (myocyte enhancer factor 2C) | 5’-AGGCAGCAAGAATACGATGC-3’  5’-TACGGAAACCACTGGGGTAG-3’ | 88 |
| MYH7  (myosin heavy chain 7) | 5’-CTGGGGTGAAAGAGAAGCGT-3’  5’-CGCAGGTTGGTCATCAGCT-3’ | 94 |
| TBX3  (T-box transcription factor 3) | 5’-CTGTAGGGACATCGAACCTCA-3’  5’-CCATGCTCCTCTTTGCTCTC-3’ | 82 |
| TBX5  (T-box transcription factor 5) | 5’-GCAGTGATGACATGGAGCTG-3’  5’-TGCTGAAAGGACTGTGGTTG-3’ | 114 |
